# Supplementary material for: Soil Collected in the Great Smoky Mountains National Park Yielded a Novel Listeria sensu stricto Species, L. swaminathanii
Source: Microbiol Spectr. 2022 Jun 6;10(3):e00442-22. doi: 10.1128/spectrum.00442-22 (PMC9241783; doi:10.1128/spectrum.00442-22)
Supplement: Supplemental file 1 — Supplemental material. Download spectrum.00442-22-s0001.pdf, PDF file, 0.4 MB [file spectrum.00442-22-s0001.pdf]

Table S1: *L. swaminathanii* sp. nov. growth following incubation at various temperatures

Strain	Growth results at each incubation time and temperature†									
	4˚C		22˚C		30˚C		37˚C		41˚C	
	10 d	14 d	24 h	48 h	24 h	48 h	24 h	48 h	24 h	48 h
L. swaminathanii FSL L7-0020^T	4.34	5.47	7.11	9.20	9.26	8.98	9.30	8.41	6.20	8.65
L. monocytogenes 10403S	2.76	4.14	6.41	9.20	9.15	8.34	8.94	8.20	8.94	8.04

†The log_{10} CFU relative growth calculated as the average of two biological replicates minus the starting inoculums for each time and temperature combination. Each biological replicate is the average of two technical replicates.

‡*L. monocytogenes* 10403S was used as a control sample

Table S2: Results from API CH50 tests not reported on Table 1

	L. swaminathanii	***L. cossartiae****
Erythritol	-	-
D-Arabinose	-	-
L-Xylose	-	-
D-Adonitol	-	-
Methyl-α-D-Xylopyranoside	-	-
D-Fructose	+	+
D-Mannose	+	+
Dulcitol	-	-
D-Sorbitol	-	-
N-Acetylglucosamine	+	+
Amygladin	+	+
Arbutin	+	+
Salicin	+	+
D-Cellobiose	+	+
D-Trehalose	-	V
D-Rafinose	-	-
Starch (amidon)	+	-
Glycogen	-	-
Xylitol	+	+
Gentiobiose	+	+
D-Fucose	-	-
L-Fucose	-	-
L-Arabitol	-	-
Potassium Gluconate	-	-
Potassium 2-Ketogluconate	-	-
Potassium 5-Ketogluconate	-	-

* *L. cossartiae* supplementary results from our previous study (21) are included to show the differentiating starch result

+ positive; - negative; V variable between replicates

Table S3: *Listeria* spp. flagellar motility genes from the cgMSLT1748 scheme

Locus	Full name/product
lmo0676	flagellar biosynthesis protein FliP
lmo0677	flagellar biosynthesis protein FliQ
lmo0678	flagellar biosynthesis protein FliR
lmo0680	flagellar biosynthesis protein FlhA
lmo0681	flagellar biosynthesis regulator FlhF
lmo0682	flagellar basal body rod protein FlgG
lmo0685	flagellar motor protein MotA
lmo0686	flagellar motor rotation MotB
lmo0690	flagellin
lmo0693	flagellar motor switch protein FliY
lmo0696	flagellar basal body rod modification protein
lmo0697	flagellar hook protein FlgE
lmo0698	flagellar motor switch protein
lmo0699	flagellar motor switch protein FliM
lmo0700	flagellar motor switch protein FliY
lmo0705	flagellar hook-associated protein FlgK
lmo0706	flagellar hook-associated protein FlgL
lmo0707	flagellar capping protein FliD
lmo0708	flagellar protein
lmo0710	flagellar basal-body rod protein FlgB
lmo0711	flagellar basal body rod protein FlgC
lmo0712	flagellar hook-basal body protein FliE
lmo0713	flagellar MS-ring protein FliF
lmo0714	flagellar motor switch protein FliG
lmo0715	flagellar assembly protein H
lmo0716	flagellum-specific ATP synthase

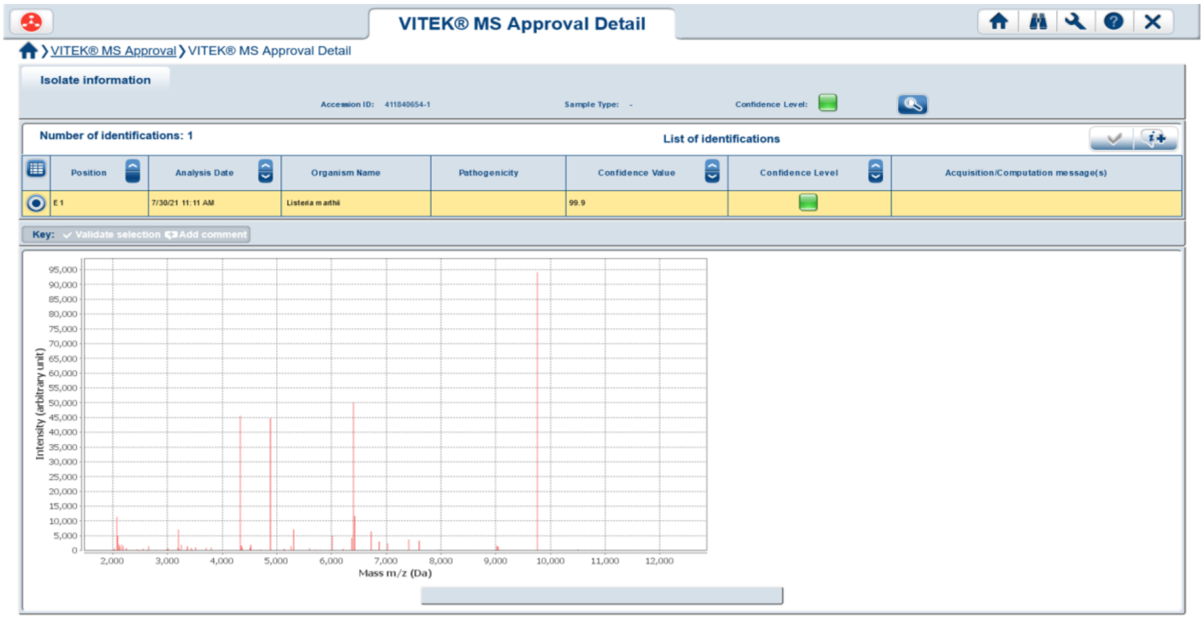

Fig. S1. The spectra file for *L. swaminathanii* FSL L7-0020[T] following analysis by VITEK MS V3.2 and the protocol for bacteria. Briefly, a fresh culture was prepared by streaking an isolated colony to trypticase soy agar (TSA) and incubated for 18-24 h at 35˚C. A portion of a colony was then used to prepare a VITEK MS-DS target slide followed by automated MALDI-TOF analysis from which the resulting spectra is compared to a database. The *Listeria* database is currently represented by strains of *L. monocytogenes*, *L. innocua, L. ivanovii, L. seeligeri, L. welshimeri*, and *L. marthii*. *L. swaminathanii* FSL l7-0020[T] was identified as *L. marthii* with a confidence value of 99.9.
